# Growth Pattern, Resting Energy Expenditure, and Nutrient Intake of Children with Food Allergies

**DOI:** 10.3390/nu11020212

**Published:** 2019-01-22

**Authors:** Enza D’Auria, Valentina Fabiano, Simona Bertoli, Giorgio Bedogni, Alessandra Bosetti, Erica Pendezza, Marco Ugo Andrea Sartorio, Alessandro Leone, Angela Spadafranca, Barbara Borsani, Francesco Stucchi, Alberto Battezzati, Gian Vincenzo Zuccotti

**Affiliations:** 1 Pediatric Department, Vittore Buzzi Children’s Hospital, Università degli Studi di Milano, via Castelvetro 32, 20154 Milan, Italy; enza.dauria@unimi.it (E.D.); alessandra.bosetti@asst-fbf-sacco.it (A.B.); erica.pendezza@unimi.it (E.P.); marco.sartorio@unimi.it (M.U.A.S); barbara.borsani@unimi.it (B.B.); francesco.stucchi@studenti.unimi.it (F.S.); gianvincenzo.zuccotti@unimi.it (G.V.Z.); 2 International Center for the Assessment of Nutritional Status (ICANS), Department of Food, Environmental and Nutritional Sciences (DeFENS), Università degli Studi di Milano, Via Sandro Botticelli 21, 20133 Milan, Italy; simona.bertoli@unimi.it (S.B.); alessandro.leone1@unimi.it (A.L.); angela.spadafranca@unimi.it (A.S.); alberto.battezzati@unimi.it (A.B.); 3 Clinical Epidemiology Unit, Liver Research Center, AREA Science park, Strada Statale 14, km 163.5, 34012 Trieste, Italy; giorgiobedogni@gmail.com

**Keywords:** resting energy expenditure, indirect calorimetry, food allergy, food intake, children

## Abstract

Growth impairment has been reported in children with food allergies (FA). However, the available data on the dietary intake of FA children are controversial, and no data are available on their resting energy expenditure (REE). The aim of this study was to test whether REE differs between FA and healthy children. In this study, 30 FA children were matched by sex and age, with 31 healthy controls using coarsened exact matching (CEM). Their REE was measured by indirect calorimetry (IC). Energy and macronutrient intake were evaluated using a three-day dietary record. Between-group comparisons were performed by robust median regression using CEM-related weights. The association of REE with allergies was also evaluated using robust median regression models. Anthropometric measurements, REE, and nutrient intake were similar in FA children and matched controls. Taking into account the association of REE with gender and age, a statistically significant but biologically negligible association was detected between median REE and allergy status (+9% in FA children). In conclusion, we did not find any biologically relevant difference in REE, anthropometry, and dietary intake in children with FA compared to healthy children.

## 1. Introduction

The prevalence of food allergies (FA) as detected by an oral food challenge is 7–8% in Europe [1] and 8–10% in the USA and Australia [2,3]. The foods most commonly involved in FA are cow’s milk, hen’s eggs, peanuts, tree nuts, and wheat [4]. The mainstay treatment of FA is the avoidance of culprit food, the so called “avoidance diet”, because eating this food may trigger symptoms and, less frequently, produce life-threatening consequences such as anaphylaxis [5].

Recently, much effort has been directed toward a personalized nutritional approach to FA, including advice to avoid unnecessary elimination diets. This is especially important in children because of their sensitivity of growth to nutritional deficiencies. The impact of FA on growth has been investigated by different studies with controversial findings. While some studies have shown growth impairment in FA infants and young children [6,7,8,9,10], other studies found no FA impact on growth [11,12,13], even in children with multiple food allergies [12]. Such discrepant results may be explained by the different case-mix of patients as well as by the provision of dietary advice given by dieticians in some studies [12,13]. Specific food effects are, however, not easy to rule out. For instance, some studies have shown that the avoidance of cow milk may negatively affect the growth of infants and young children [9,14]. However, a recent retrospective study found no difference in the growth of FA and healthy children, irrespective of the number of foods eliminated, except for the children suffering from cow’s milk allergy (CMA) [15]. The effect of atopic co-morbidities on growth and food avoidance has been addressed by only a few studies, focusing mostly on atopic dermatitis [16,17] and asthma [18,19,20,21]. Contrasting findings have also been reported on the nutrient intake of FA vs. healthy children, with some studies showing lower energy and protein intake in the former [6,9,22], and other studies showing growth impairment in the former, with energy and protein intake similar to that of the latter [8]. Different hypotheses have been put forward to explain such findings, including nutrient loss due to sustained intestinal or skin inflammation despite the avoidance diet [6,12,23]. The effective nutritional requirements of FA children are presently unknown.

One of the major challenges of modern clinical nutrition is the implementation of tailored nutritional recommendations. In this view, the first step toward a personalized nutritional approach is a reliable knowledge of the patient’s energy expenditure. The reference method for measuring resting energy expenditure (REE) is indirect calorimetry (IC) [24,25]. However, IC is often not available in clinical practice because of its cost and the need for trained personnel [26]. For these reasons, REE is often estimated by predictive equations. Such equations are easy to use but have large individual errors and must be employed with caution [27]. Importantly, for the tailored nutritional approach, no study so far has measured REE by IC in FA children.

The aim of this study was therefore to measure REE by IC in FA children and compare it to that of sex- and age-matched children.

## 2. Materials and Methods

### 2.1. Study Design

We performed a case-control study where children with FA (“cases”) were matched by sex and age to healthy children (“controls”).

The FA children were enrolled between May 2017 and October 2018, and were selected from the children who were referred to the allergy unit of the Vittore Buzzi Children’s Hospital (Milano, Italy) because of suspected FA. Their FA were definitively diagnosed by an oral food challenge in children with suggestive clinical history and positive prick tests or food-specific immunoglobulins, with the exception of those children with known anaphylaxis. The FA children were eligible for the study if they (1) had a diagnosis of FA by oral food challenge, (2) had been on an avoidance diet for at least 6 months and, (3) were able to perform IC.

Control children were selected from a larger sample of children recruited from the general population between April 2017 and October 2018. Control children were eligible for the study if they (1) did not have FA, (2) were free of known acute (e.g., influenza) or chronic (e.g., diabetes) diseases, and (3) were able to perform IC.

All children underwent IC and anthropometry, and their parents were given a prospective three-day food dietary record as described below.

The study was performed in accordance with the Declaration of Helsinki, and the parents of each child gave their written informed consent. The study protocol was approved by the local ethical committee (2018/ST/267).

### 2.2. Resting Energy Expenditure

We used an open-circuit ventilated-hood calorimeter (Sensor Medics 29, Anaheim, CA, USA) to measure oxygen consumption (VO_2_) and carbon dioxide production (VCO_2_). Such measurements were taken in a thermoneutral environment (ambient temperature 24 to 26 °C) devoid of external stimuli. At the beginning of each test, the calorimeter was calibrated with two different gas mixtures (26% O_2_ and 74% N_2_; 16% O_2_, 4% CO_2_ and 80% N_2_). The children had been fasting for at least 8 h and had had a stable respiratory function for at least 1 h before performing IC. The data collection time was at least 20 min. A run-in time of at least 5 min was used to obtain stable measures and to allow the children to get used to the canopy and the instrument noise. The presence of the steady state was determined by 5 consecutive minutes in which VO_2_ and VCO_2_ variations were less than 10%. Their REE was estimated using the Weir equation [28].

### 2.3. Anthropometric Measurements

Anthropometric measurements were collected by trained dietitians following standard guidelines [29]. Body weight was measured to the nearest 100 g with a beam scale, and body height to the nearest 0.1 cm using a vertical stadiometer. BMI was calculated as weight (kg)/height (m)^2^. The standard deviation scores (SDS) of weight, height, and BMI were calculated using WHO reference data. Biceps, triceps, subscapular, and suprailiac skinfolds were measured using a Tanner-Whitehouse caliper (Holtain Ltd., UK). Each skinfold was measured three times, and the mean value was used for analysis.

### 2.4. Nutrient Intake

A prospective three-day food dietary record was used to measure nutrient intake. The parents were trained by a dietitian on how to fill in the diary of two working days and one weekend day. Each record consisted of one page, including three main meals (breakfast, lunch, and dinner) and two snacks (mid-morning and mid-afternoon). The parents were asked to report the food consumed; its quantity; and the time of its consumption, including a detailed description (e.g., brand name, ingredients used, recipe, and cooking method) and the amount of each item. They were asked to weigh each single food item, or each ingredient of a homemade recipe, as well as leftovers and to record all the weights in the food diary. When food or beverage weighing was not possible, parents were instructed to use common instruments, such as spoons, graduated cups, or bowls, to quantify them. Energy and macronutrient intake (protein, carbohydrates, and fats) were estimated from the three-day food data using the Metadieta software (Me.Te.Da. S.r.l., San Benedetto Del Tronto, Italy).

### 2.5. Statistical Analysis

Coarsened exact matching (CEM) was used to match cases to controls on the basis of sex (same) and age (within 1 year) [30]. Descriptive statistics of continuous variables were reported as percentiles because most of them had non-Gaussian distributions.

Between-group comparisons were performed by robust median regression (quantile regression of the 50th percentile) using CEM-related weights [31]. We investigated the association of REE with allergies by using three pre-specified median regression models:

Model (1) an univariable model using allergies as the predictor (discrete; 0 = no, 1 = yes);

Model (2) a multivariable model adding sex (discrete; 0 = female, 1 = male) and age (continuous, years) to the predictor of Model 1 and;

Model (3) a multivariable model adding weight (continuous, kg) to the predictors of model 2.

Model 1 tested whether there is a difference in REE between allergic and non-allergic children; model 2 evaluated the contribution of age and gender independently from allergies; and, lastly, model 3 tested whether allergies added to the most commonly employed predictors of REE, i.e., sex, age, and body weight. The linearity of the association between REE and continuous predictors was tested using fractional polynomials [32]. As post-hoc analyses, we performed quantile regressions of the 25th and 75th percentiles, using the same predictors utilized for the 50th percentile regression (see above). All regression analyses took CEM into account by using CEM-related weights and robust 95% confidence intervals. Statistical analysis was performed using Stata 15.1 (Stata Corporation, College Station, TX, USA) together with the user-written CEM command [33].

## 3. Results

Using CEM, we matched 30 FA children with 31 healthy children.

Among the FA children, 10 were allergic to one food, six to two foods, and 14 to more than two foods. The foods most commonly responsible for FA were tree nuts (*n* = 20), eggs (*n* = 12), cow’s milk (*n* = 11), fish (*n* = 9), fruits (*n* = 7), peanuts (*n* = 5), legumes (*n* = 4), sesame (*n* = 2), and vegetables (*n* = 1). Among the FA children, six had ongoing and five had previous atopic dermatitis, while nine had ongoing and one had previous asthma.

Table 1 gives the distribution of sex and BMI in control and FA children.

Table 2 gives the anthropometric measurements, REE, and the nutrient intake of FA and control children.

The anthropometric measurements, REE, and nutrient intake were similar in FA and control children (median regression, *p* > 0.05 for all comparisons).

Table 3 gives the 50th percentile (median) regression models that were used to investigate the association between REE and food allergies.

No association between FA and energy intake was apparent from Model 1. Taking into account the expected association of REE with gender and age as done by Model 2, a statistically significant but biologically negligible association between allergies and REE becomes evident (8% of total REE calculated as 88/1107 kcal, where the denominator is the REE of the whole sample). A similar difference persisted after the expected association with weight was taken into account by Model 3 (9% calculated as 99/1107 kcal, where the denominator is the REE of the whole sample).

Table 4 gives the 25th percentile regression models used to investigate the association between REE and food allergies.

There was no statistically significant association between REE and allergies in any of the models. According to Model 3, allergies accounted for 1% of REE, calculated as 13/950 kcal, where the denominator is the REE of the whole sample.

Table 5 gives the 75th percentile regression models used to investigate the association between REE and food allergies.

According to Model 3, allergies accounted for 9% of REE, calculated as 115/1243 kcal, where the denominator is the 75th percentile REE of the whole sample.

## 4. Discussion

In this matched case-control study, we found no biologically relevant difference in REE, anthropometry, and nutrient intake of FA compared to control children. It must be pointed out that our FA children were given dietary advice according to Italian dietary guidelines [34] and were regularly followed-up, which may explain why their anthropometric status and nutrient intake did not differ from that of healthy controls. However, our data did not support the hypothesis that REE may differ in FA compared to control children because of the underlying disease.

FA are defined as “an adverse health effect arising from a specific immune response that occurs reproducibly on exposure to a given food” [35] and encompass a range of disorders from IgE-mediated anaphylaxis to delayed cell-mediated reactions affecting the gastrointestinal tract. The mainstay treatment of FA is the avoidance diet. Food exclusion is highly effective but impairs the quality of life of FA patients and puts them at risk of eating an unbalanced diet. A tailored dietary management of FA children is the key to ensure adequate growth [4]. Such management includes the evaluation of REE, which is the main determinant of energy expenditure in physiological conditions [27].

This study is the first one to measure REE in FA compared to healthy children. Our data did not support the hypothesis that REE may be increased in FA children because of the underlying disease, e.g., by the underlying inflammatory status. Albeit statistically significant, the estimate of 9% of REE attributable to allergies, obtained by a multivariable model based on gender, age, and weight, is too low to be biologically relevant, being very close to the measurement error of IC.

Our study had some limitations. The first limitation was its small sample size. This is partly explained by the strict matching criteria, which were nonetheless a point of strength of this study. Despite the strict matching by gender and age, gender and age actually contributed to REE independently from allergies. A second limitation of this study was the lack of data on the physical activity of the children, even if the median SDS of weight and BMI were well within normal limits, and being overweight and obese were similarly common in FA and healthy children. A third limitation was the lack of data on children aged ≤4 years, owing to the fact that is very difficult to perform IC on such children in a reproducible manner. Because most studies of FA were performed on infants and children younger than ours, a direct comparison with previous data may not be possible [6,7,8,10].

In contrast with previous studies showing growth impairment in children with FA [6,7,8,9,10], we did not observe biologically important differences in anthropometry and nutrient intake between FA and control children. In particular, protein intake was similar in FA and healthy children. These results are in keeping with a previous study of growth and nutrient intake [36] and with previous growth studies [9,12] of FA children. Our FA children were being regularly followed-up and they were given dietary advice following the Italian dietary guidelines [34], which may explain why their anthropometric status and nutrient intake did not differ from that of healthy controls. The same finding was reported by other studies of FA vs. non-FA children [9,12].

As is standard in our clinical practice, the parents of the enrolled children were instructed not only about the foods to avoid but also how to replace them and to avoid unnecessary exclusion diets. For instance, except for one child, all children with CMA were allowed to eat beef, if they were prick by prick negative, because most CMA children can tolerate beef [37]. Likewise, given the low cross-reactivity among legumes, children with clinical evidence of reactivity to peanuts were advised not to routinely avoid other legumes if the corresponding sIgE were negative. When sIgE were positive for legumes, an oral food challenge was performed to exclude a concomitant legume allergy [38]. Even though our understanding of cross-reactivity between different foods is rapidly increasing, unnecessary elimination diets are still very common in clinical practice [39]. Moreover, the diet of CMA children was, in all but three cases, expanded by including heated milk, which allowed them to consume selected baked products and snacks increasing their energy intake [40,41]. These and other dietary personalization strategies are central to the management of FA [42,43].

## 5. Conclusions

In conclusion, in FA children regularly followed with personalized dietary advice, we did not find any difference in REE, anthropometry, and dietary intake as compared to sex- and age-matched control children. Because this is the only study of the REE of FA children available so far, further studies are certainly needed to better understand energy expenditure in FA.

## Figures and Tables

**Table 1:** Distribution of sex and body mass index (BMI) of the children.

	Controls (*n* = 31)		Food Allergies (*n* = 30)	
	*n*	%	*n*	%
**Sex**				
Female	18	56.7	17	56.7
Male	13	43.3	13	43.3
Total	31	100.0	30	100.0
**BMI class according to World Health Organization (WHO) averages**				
Underweight	1	3.9	1	3.3
Normal weight	25	82.1	23	76.7
Overweight	0	1.3	3	10.0
Obese	4	12.8	3	10.0
Total	31*	100.0	30	100.0

* Partials do not sum to 31 because of rounding (matching strata).

**Table 2:** Anthropometric measurements, resting energy expenditure (REE), and nutrient intake of the children.

	Controls (*n* = 31)			Food Allergies (*n* = 30)		
	P_25_	P_50_	P_75_	P_25_	P_50_	P_75_
Age (years)	6	7	9	6	7	9
Weight (kg)	20.7	23.7	37.9	21.2	25.1	31.0
Weight (SDS WHO)	−0.65	0.16	0.74	−0.73	0.08	0.78
Height or length (m)	1.20	1.24	1.38	1.18	1.26	1.34
Height (SDS WHO)	−0.16	0.62	1.17	−0.76	0.14	0.83
BMI (kg/m^2^)	14.7	15.4	16.1	14.8	15.7	16.9
BMI (SDS WHO)	−1.01	−0.08	0.31	−0.62	-0.25	0.69
Triceps skinfold (mm)	8	9	12	9	11	14
Biceps skinfold (mm)	5	6	7	6	7	10
Subscapular skinfold (mm)	5	5	6	5	6	8
Suprailiac skinfold (mm)	5	6	10	4	6	10
Sum of 4 skinfolds (mm)	26	28	34	25	30	45
REE (kcal/day)	926	1073	1180	984	1103	1251
REE (kcal/kg weight/day)	33	43	45	37	44	49
Energy (kcal/day)	1313	1490	1733	1419	1712	2056
Energy (kcal/kg weight/day)	57	65	72	55	63	79
Proteins (g)	44	52	64	45	56	70
Fats (g)	44	58	69	54	64	71
Carbohydrates (g)	167	197	229	172	226	252
Proteins (% Energy)	12.6	13.3	14.7	12.4	13.8	15.3
Fats (% Energy)	30.0	31.3	35.9	30.3	34.0	38.1
Carbohydrates (% Energy)	49.6	55.6	56.4	47.4	52.6	56.0
Proteins (g/kg weight/day)	1.8	2.1	2.5	1.8	2.2	2.7

Abbreviations: P_25_ = 25th percentile; P_50_ = 50th percentile (median); P_75_: 75th percentile; SDS = standard deviation score; WHO = World Health Organization; BMI = body mass index; REE = resting energy expenditure.

**Table 3:** Association between food allergies and resting energy expenditure (REE): 50th percentile (median) regression equations.

	Model 1 (kcal/day)	Model 2 (kcal/day)	Model 3 (kcal/day)
Food allergies (yes)	34 [−132 to 200]	88 * [14 to 163]	99 ** [29 to 168]
Age (years)		59 *** [42 to 77]	12 * [0 to 24]
Sex (male)		114 * [27 to 200]	94 *** [41 to 147]
Weight (kg)			13 *** [10 to 17]
Intercept	1073 *** [939 to 1207]	513 *** [327 to 698]	553 *** [423 to 682]
*N*	61	61	61

Abbreviations: * *p* < 0.05, ** *p* < 0.01, *** *p* < 0.001. Values are medians and robust CEM-weighted 95% confidence intervals (in brackets). As an instance of how to predict REE using these equations, consider the case of a seven-yr-old allergic boy weighing 25 kg. Using Model 3, his median REE can be estimated as follows: (99 × 1) + (12 × 7) + (94 × 1) + (13 × 25) + 553 = 1155 kcal. (Note that these equations take the matching of FA and control children into account via matching-related weights, see statistical analysis for details).

**Table 4:** Association between food allergies and resting energy expenditure (REE): 25th percentile regression equations.

	Model 1 (kcal/day)	Model 2 (kcal/day)	Model 3 (kcal/day)
Food allergies (yes)	58 [−111 to 227]	37 [−92 to 165]	13 [−85 to 111]
Age (years)		68 *** [42 to 94]	23 [−2 to 47]
Sex (male)		120 [−5 to 246]	81 ** [20 to 141]
Weight (kg)			14 *** [9 to 19]
Intercept	926 *** [823 to 1029]	402 ** [153 to 650]	410 *** [252 to 569]
*N*	61	61	61

Abbreviations: ** *p* < 0.01, *** *p* < 0.001. Values are 25th percentiles and robust CEM-weighted 95% confidence intervals (in brackets).

**Table 5:** Association between food allergies and resting energy expenditure (REE): 75th percentile regression equations.

	Model 1 (kcal/day)	Model 2 (kcal/day)	Model 3 (kcal/day)
Food allergies (yes)	71 [−123 to 265]	69 [−49 to 187]	115 ** [45 to 185]
Age (years)		49 ** [20 to 79]	9 [−29 to 46]
Sex (male)		26 [−86 to 138]	102 * [18 to 186]
Weight (kg)			13 * [1 to 26]
Intercept	1180 *** [1033 to 1327]	729 *** [496 to 963]	595 *** [381 to 810]
*N*	61	61	61

Abbreviations: * *p* < 0.05, ** *p* < 0.01, *** *p* < 0.001. Values are 75th percentiles and robust CEM-weighted 95% confidence intervals (in brackets).
